# ECO-CollecTF: A Corpus of Annotated Evidence-Based Assertions in Biomedical Manuscripts

**DOI:** 10.3389/frma.2021.674205

**Published:** 2021-07-13

**Authors:** Elizabeth T. Hobbs, Stephen M. Goralski, Ashley Mitchell, Andrew Simpson, Dorjan Leka, Emmanuel Kotey, Matt Sekira, James B. Munro, Suvarna Nadendla, Rebecca Jackson, Aitor Gonzalez-Aguirre, Martin Krallinger, Michelle Giglio, Ivan Erill

**Affiliations:** ^1^ Department of Biological Sciences, University of Maryland Baltimore County, Baltimore, MD, United States; ^2^ Institute for Genome Sciences, University of Maryland School of Medicine, Baltimore, MD, United States; ^3^ Barcelona Supercomputing Center (BSC), Barcelona, Spain; ^4^ Centro Nacional de Investigaciones Oncológicas (CNIO), Madrid, Spain

**Keywords:** evidence, annotation, corpus, text- and data mining, literature, biocuration

## Abstract

Analysis of high-throughput experiments in the life sciences frequently relies upon standardized information about genes, gene products, and other biological entities. To provide this information, expert curators are increasingly relying on text mining tools to identify, extract and harmonize statements from biomedical journal articles that discuss findings of interest. For determining reliability of the statements, curators need the evidence used by the authors to support their assertions. It is important to annotate the evidence directly used by authors to qualify their findings rather than simply annotating mentions of experimental methods without the context of what findings they support. Text mining tools require tuning and adaptation to achieve accurate performance. Many annotated corpora exist to enable developing and tuning text mining tools; however, none currently provides annotations of evidence based on the extensive and widely used Evidence and Conclusion Ontology. We present the ECO-CollecTF corpus, a novel, freely available, biomedical corpus of 84 documents that captures high-quality, evidence-based statements annotated with the Evidence and Conclusion Ontology.

## Introduction

Life scientists have become increasingly dependent on the availability of standardized scientific information in order to infer new knowledge from high-throughput experiments (Marx, 2013; Reshetova et al., 2014). This standardized knowledge derives largely from information extracted by expert curators from journal articles (Hirschman et al., 2012; Verspoor et al., 2012; Kwon et al., 2018). Because curators cannot keep pace with the volume of articles published, automated text mining plays an important role in curation (Hirschman et al., 2012; Verspoor et al., 2012; Kwon et al., 2018; Kim et al., 2008; Islamaj Dogan et al., 2017a). Biomedical text mining has incorporated a diverse palette of machine learning techniques (Aggarwal and Zhai, 2012; Jovanović and Bagheri, 2017; Wei et al., 2019; Chen et al., 2020; Zhang et al., 2019; Lee et al., 2019). It is widely recognized that manually-constructed, gold standard biomedical corpora are key resources for the development of biomedical text mining systems, enabling the training and tuning of text mining methods to obtain optimal performance (Verspoor et al., 2012; Islamaj Dogan et al., 2017a; Wei et al., 2019; Zhang et al., 2019; Chen et al., 2020).

Standardized scientific information and controlled vocabularies define relations between biological entities of interest or to their roles, characteristics or biological attributes. For instance, one type of annotation important for biomedical knowledge discovery links gene products to their molecular functions, biological processes, and cellular locations as defined in the Gene Ontology (GO) (Barrell et al., 2009). During annotation, curators examine sentence-level statements in journal articles and use the sentences, along with the associated experimental evidence, to create annotations (Chibucos et al., 2014a; Doğan et al., 2014). Capture of supporting experimental evidence allows readers and algorithms to gauge the reliability of annotation statements and is thus crucial for enhancing the confidence of the information extracted from the text during curation (Chibucos et al., 2014a; Clark et al., 2014). A corpus of standardized biomedical knowledge must, therefore, annotate evidence-based statements. These statements must contain both assertions and clear references to the evidence backing them. Although efforts have been made to promote and evaluate the detection of experimental evidence (Krallinger et al., 2008), there is a clear need to generate more comprehensive resources and text annotation schemes for experimental evidence information.

In this work, we present the ECO-CollecTF corpus, a novel biomedical corpus capturing high-quality annotation of sentences from publications that specifically describe evidence for biological assertions. These evidence annotations are captured using the Evidence and Conclusion Ontology (ECO) (Giglio et al., 2019), a comprehensive set of terms describing evidence types and the relationships between them. In ECO, evidence is defined not simply as a technique, but as the use of a technique to enable the assertion of a conclusion. The ECO-CollecTF corpus uses the ECO evidence terms to provide annotations for evidence-based assertions: statements that make an assertion that relies on explicitly stated evidence. Many corpora have been created to satisfy a range of goals (IslamajDogan et al., 2017a; Doğan et al., 2014; Pyysalo et al., 2007; Rebholz-Schuhmann et al., 2007; Vincze et al., 2008; Gerner et al., 2010; Bada et al., 2012; Pafilis et al., 2013; Van Auken et al., 2014; Pyysalo et al., 2015; Hicks et al., 2018; Ohta et al., 2012) but only a handful of corpora have included annotation of evidence as part of the curation process. In Rzhetsky et al. (2009) Rzhetsky and co-workers annotated evidence using a self-defined set of four evidence categories. Other work has made use of GO evidence codes, which map to top-level terms in ECO and therefore involve substantial generalization in the mapping process (Crangle et al., 2007; Van Auken et al., 2014). Recently, annotation of microbial phenotypes in journal articles has been undertaken using ECO terms, but the resulting corpus is not yet available (Siegele et al., 2019). Hence, to date no available corpus provides annotations specifically to statements about evidence and which draw from the extensive range of evidence terms in ECO.

The release of the ECO-CollecTF corpus reported here contains 84 documents. Each document in the corpus was annotated independently by at least three curators, following the guidelines and training materials available in this release. The corpus has been developed to support FAIR principles (Wilkinson et al., 2016). It is available in BRAT (Stenetorp et al., 2012), a *de facto* standard format for biomedical corpora, as well as in BioC (Comeau et al., 2013), a format developed for biomedical text mining interoperability, prompted by the BioCreative initiative. A permanent, open access and freely available version of the corpus is accessible via the ECO website (http://evidenceontology.org/annotation_resources/) and deposited in Zenodo for persistence (DOI: 10.5281/zenodo.4568935).

Furthermore, unlike the majority of other biomedical corpora, we provide the annotations from each curator separately instead of a consensualized corpus. The arrangement allows other researchers to use the individual annotations to generate a consensualized corpus in whatever manner they choose. It also allows researchers to independently assess the consistency of the corpus using their Inter-Annotator Agreement (IAA) measure of choice. Given the intrinsic link of our corpus with ECO, here we introduce, deploy and empirically categorize a modification of Cohen’s *K* IAA metric (Cohen, 1960) that assigns information theoretical weights to ontology nodes to evaluate disagreement in a principled manner. In addition, we leverage the corpus development to enhance ECO by providing examples of use from selected annotations. Hence, this work puts forward a corpus that is innovative in terms of its target subject, its tight integration with the target ontology, the availability of all curator annotations, and the use and characterization of a novel metric for IAA assessment in ontology-based annotation settings.

## Materials and Methods

### Preliminary Curation Review and Scope

Annotating evidence statements associated with biological assertions in scientific text is a complex task predicated by the need to properly define the entities that must be annotated and to adequately narrow the scope of the annotation into a feasible process. In order to define what would be annotated and to scope the task, we conducted a preliminary review and annotation of three set-aside articles.

The preliminary review resulted in a collection of examples, a set of guidelines and training documents, and the definition of the curation process, which is discussed further below. The three set-aside articles used for the review were retained as training documents for curators joining the team.

### Guidelines and Annotation Schema

**FIGURE 1:**
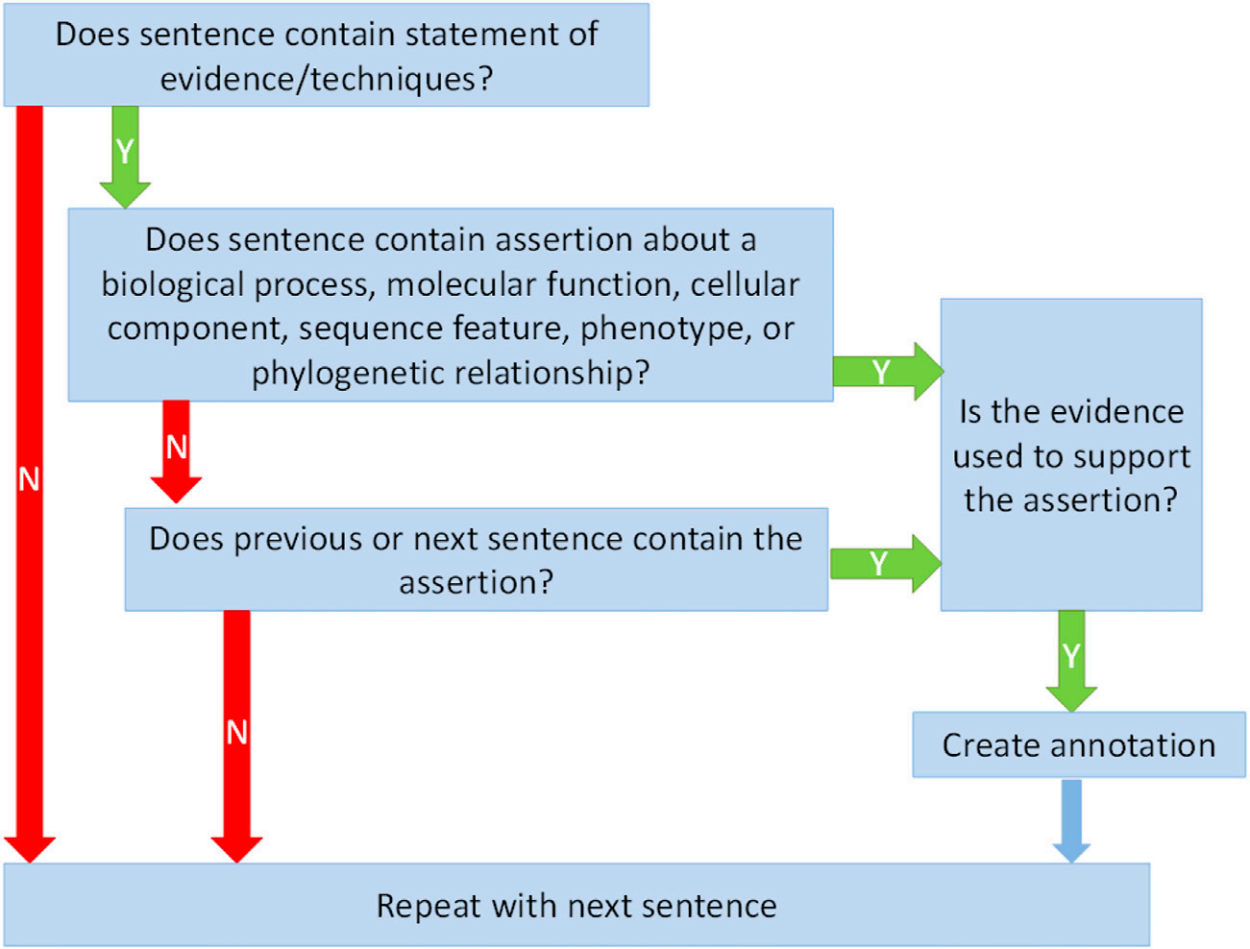
Schematic of the evidence statement annotation process. Boxes indicate question-based steps in the annotation process. Arrows show the alternative flow-paths. Only evidence statements that are used to support a specific set of types of assertions are annotated.

This section describes the annotation guidelines (Supplementary Material 1). The guidelines outline the basic annotation process (Figure 1) and identify the main elements of the annotation: sentence selection, evidence types, assertion objects, and annotation qualifiers.

#### Sentence Selection

In order to construct a practically exploitable resource we have constrained the corpus to the annotation of ECO terms when the evidence description and its use to make a claim about some entity are clearly stated and self-contained within the sentence or within an adjacent pair of sentences. As such, the following requirements must be met:

• Based on the local context, we can determine that an evidence description is being made.
• Within the same local context (sentence or pair of consecutive sentences), some assertion is made based on the evidence. That is, the authors make a claim about some entity, and it is explicitly stated or evident that the claim is made based on the evidence.

**FIGURE 2:**
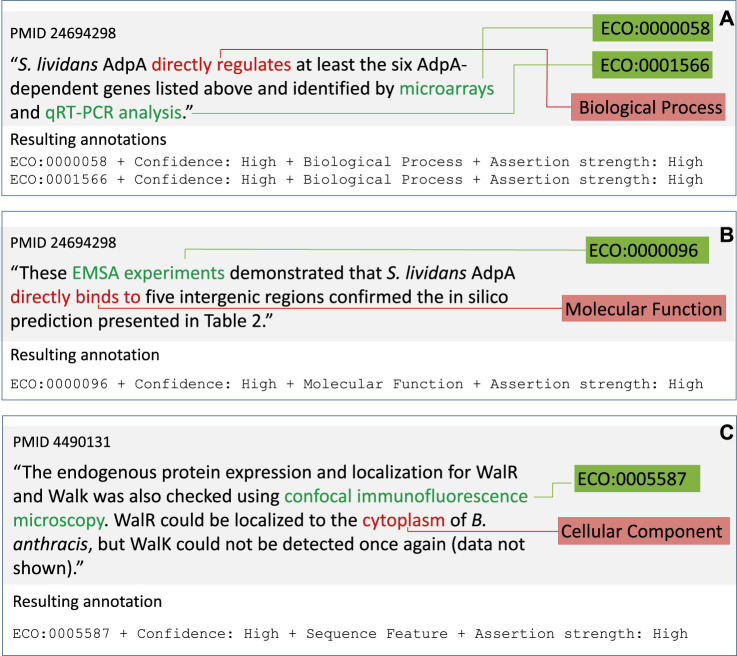
Example gene product annotations. **(A)** Multiple annotations in one sentence with Biological Process category. **(B)** Annotation with Molecular Function category. **(C)** Annotation with Cellular Component category. Text segments in the sentence mapping to ECO terms (green boxes) are highlighted in green. Text segments indicating the category of assertion (red boxes) are highlighted in red. The ECO term, ECO mapping confidence, Category, and Assertion Strength are displayed underneath the annotated text.

Each occurrence of evidence and assertion in a sentence, or pair, is given a separate annotation. Hence, multiple annotations can result from a single sentence or sentence pair as shown in Figure 2A.

#### Annotation Categories of Interest

We only annotate when the type of entity about which something is being asserted is one of the following categories of interest.

**FIGURE 3:**
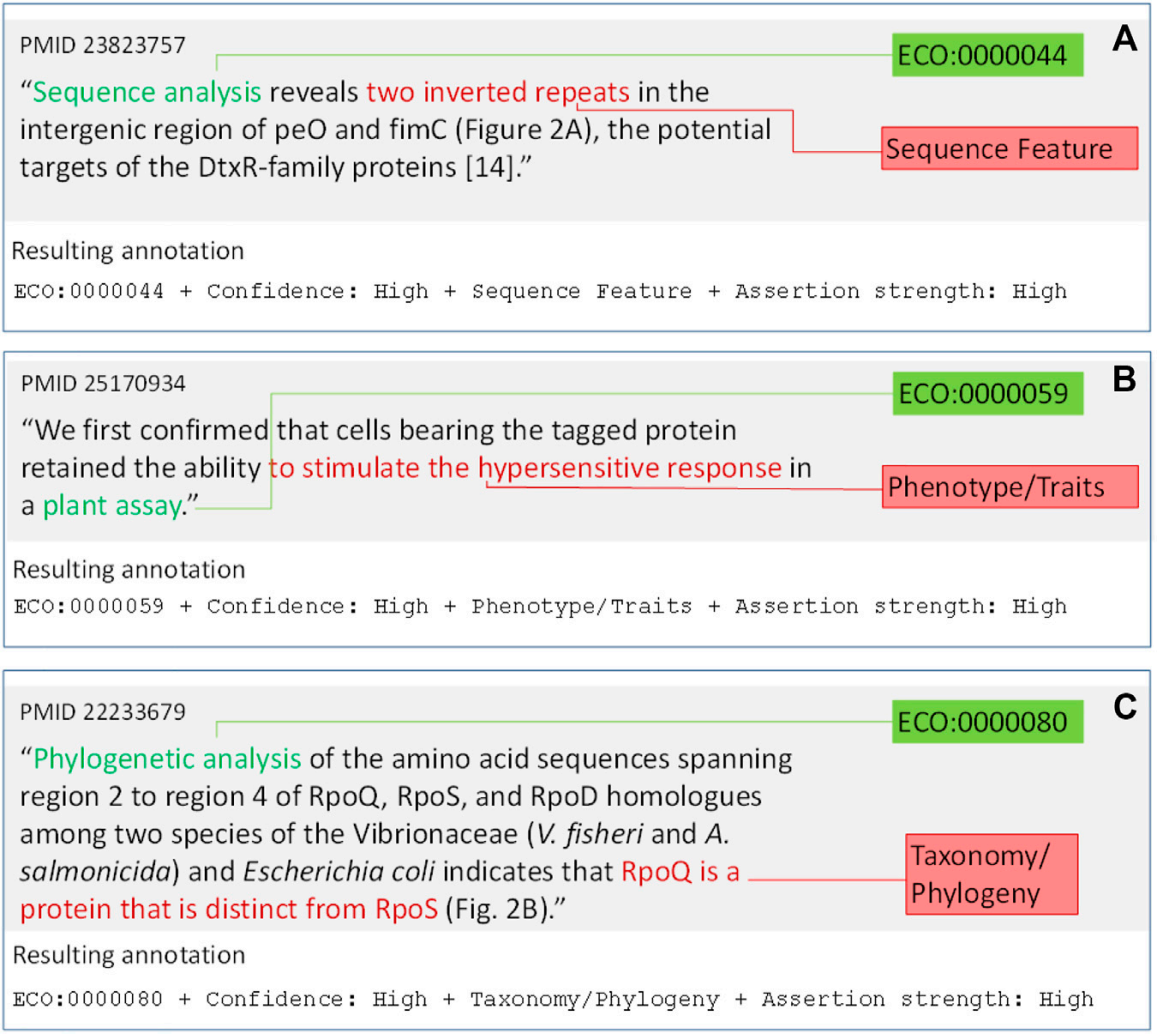
Example annotations with non-gene product annotations. **(A)** Annotation with Sequence Feature category. **(B)** Annotation with Phenotype/Traits category. **(C)** Annotation with Taxonomy/Phylogeny category. Text segments in the sentence mapping to ECO terms (green boxes) are highlighted in green. Text segments indicating the category to annotate (red boxes) are highlighted in red. The ECO term, ECO mapping confidence, Category, and Assertion Strength are displayed underneath the annotated text.

• Gene product. The entity of the assertion is the product of a gene–a protein or RNA. Usually these are the subjects of an action or a location. There are three categories of interest for gene products, based on the three sub-ontologies of the Gene Ontology.
  • Biological process (GO sub-ontology Biological Process) (Figure 2A).
  • Molecular activity (GO sub-ontology Molecular Function) (Figure 2B).
  • Location (GO sub-ontology Cellular Component) (Figure 2C).
• Biological sequence features. The entity is a DNA, RNA, or protein sequence feature (e.g. a promoter element, protein domain, or chromosomal origin of replication) (Figure 3A). For reference we use the Sequence Ontology (Eilbeck et al., 2005) *sequence_feature* class.
• Phenotypes and traits. The entity being asserted is a phenotype (e.g. the ability to grow on acetate as a carbon source) (Figure 3B). For reference, we use the Ontology for Microbial Phenotypes (Chibucos et al., 2014b).
• Taxonomic and phylogenetic objects. What is being asserted is a taxonomic assignment (e.g. identifying the order a species belongs to, or a phylogeny-based statement about a gene, such as orthology) (Figure 3C).

#### Annotation Attributes

When an annotation is created, the following annotation attributes are assigned values by the curator.

• ECO term identifier. For example, ECO:0001566, ‘quantitative reverse transcription polymerase chain reaction evidence’, for the evidence in the sentence.
• Sentence pair. True if the annotation is for a consecutive sentence pair, false otherwise.
• Negative assertion. True if the assertion is a negative statement (e.g., “our data does not support”), false otherwise.
• ECO term selection confidence. High, Medium, or Low. This attribute captures the curator’s belief that a particular ECO term is referred to in the sentence.
• Assertion strength. High, Medium, or Low. This attribute captures the curator’s assessment of the strength of the claim made by the authors (e.g., the use of the word “conclude” would indicate a high assertion strength, while the use of the word “possibly” would indicate low assertion strength).

### Document Acquisition

We selected 87 open-access journal articles from CollecTF (Kiliç et al., 2014), a database of transcription factor binding sites (TFBS) in bacteria. We downloaded the documents from PubMed Central (Roberts, 2001) in XML format using the EFetch utility from NCBI (Sayers and Miller, 2014). We set aside three documents of different lengths and TFBS topics for the preliminary curation review; the remaining 84 documents were designated for the corpus. We developed Python scripts to process the articles into a form suitable for annotation. The XML files were parsed to extract only the results or results/discussion sections, removing any HTML tags. Greek, Latin, and other non-ASCII characters were mapped to ASCII text, as were HTML special characters. The resulting texts were tokenized and broken into sentences using the Python Natural Language Toolkit (NLTK) (Bird et al., 2009). Each result section was written to an individual plain text file with each sentence on an individual line. All scripts used to prepare the texts and instructions are available at https://github.com/ErillLab/ECO-CollecTF/tree/master/code/PrepFiles. The 84 text files were copied onto a BRAT server for annotation.

### Annotation Tool Setup

The software for the BRAT annotation tool (Stenetorp et al., 2012) is freely available at the BRAT website, http://brat.nlplab.org/. We used the current version of BRAT, v1.3 “Crunchy Frog”. The September 14, 2018 version of ECO was downloaded from http://www.evidenceontology.org/in OBO format, and converted to BRAT format using the Python script obo_to_brat_format.py, available as part of the BRAT installation. We installed ECO in BRAT using the Python script norm_db_init.py, provided in the BRAT installation. We modified three BRAT configuration files, following the instructions at the BRAT website: annotation. conf, tools. conf, and visual. conf. These files are available at https://github.com/ErillLab/ECO-CollecTF/tree/master/config.

### Curation Process

Curators were trained in the process of annotation by having them apply the established guidelines (as outlined above) to the three publications on which the guidelines were developed. They were provided with a BRAT tutorial (Supplementary Material 2) and an overview of ECO and how to browse it for needed terms. To foster direct interaction and resolution of potential discrepancies or doubts, curators were able to ask questions of the annotation coordination team throughout the process. The annotation coordination team also provided feedback and corrections to make sure the curators understood what to annotate, how to fill in the attributes, and how to use BRAT.

During the practical annotation phase, we assigned each curator a set of documents to be annotated using the BRAT tool. Curators carried out the annotation process independently. The inconsistency resolution and guideline refinement team met weekly to discuss and resolve the annotation of difficult sentences. During the meetings that occurred early in the curation effort, it became clear that some alternative interpretations of complex annotation scenarios had to be addressed, in particular regarding whether some author statements constituted evidence-based assertions or not. Therefore, we carried out an annotation refinement and retraining of curators, and updated the guidelines and examples.

In order to allow examining alternatively valid annotations, and to provide transparency with respect to these differences, the individual curator annotation results were kept separately. This enabled end users of the ECO-CollecTF corpus to exploit them according to their needs, for example, by generating a harmonized corpus though approaches such as majority voting or by comparing their system to each individual human annotator.

The 84 documents were not all annotated by the same group of curators, but rather by three different cohorts of curators at different times working on three separate subsets of documents; no documents were shared between cohorts. All curators received the same training and followed the same guidelines and process, and all documents were annotated by at least three curators.

### Inter-Annotator Agreement

Among the curators, IAA was calculated for each pair of curators who annotated the same set of documents. Four curators participated in cohort one; two curators split the documents to annotate between them. Thus, each of the 45 documents were annotated by three people, resulting in five curator pairs for cohort one. Cohort two consisted of three curators who each annotated all nine documents, leading to three curator pairs for cohort two. Four curators formed cohort three, and each of the 30 documents were annotated separately by each curator, giving six curator pairs for cohort three. Thus, in total there were 14 pairs of curators who annotated the same set of documents. The IAA was computed as described below for the 14 pairs, and these 14 IAA scores were averaged to give an overall IAA score for the corpus. Hence, the IAA values provided here apply to the entire corpus, and not to a subset of documents annotated by multiple curators, generating a more accurate estimate of inter-annotator agreement.

For sentence-level agreement (i.e. annotated or not), Cohen’s *Κ* (Cohen, 1960) was used. This is defined by Eq. 1, in which *p*_*o*_ is the observed proportion of agreement between the two curators, and *p*_*e*_ is the expected proportion of agreement based on each curator’s proportion of annotated sentences (Supplementary Material 3).

$$K=\frac{p_{o}-p_{e}}{1-p_{e}}$$ (1)

Our curation process allows annotating single or “paired” consecutive sentences. This requires that annotations to single and consecutive sentences be aligned for calculating Cohen’s *K*. For IAA computation, consecutive sentences were considered as independently annotated sentences, and annotations to each sentence by each curator were tallied separately.

### IC Inter-annotator Agreement, KwIC

Cohen’s *K* only accounts for binary agreement of whether both curators annotated a sentence or not. To calculate agreement in which the similarity of the ECO terms chosen is also taken into account, Cohen’s weighted *K* for agreement (Cohen, 1968) (K_w-agree_) was used, Eq. 2. (Supplementary Material 5)

$$K_{w-agree}=\frac{\sum w_{ij}p_{oij}-\sum w_{ij}p_{eij}}{w_{max}-\sum w_{ij}p_{eij}}$$ (2)

where *w*_*ij*_ is the weight associated with the agreement between concepts *i* and *j*, *w*_*max*_ is the largest possible agreement weight, *p*_*oij*_ is the observed proportion of annotation pairs containing concepts *i* and *j*, and *p*_*eij*_ is the expected proportion of annotation pairs containing concepts *i* and *j*.

**FIGURE 4:**
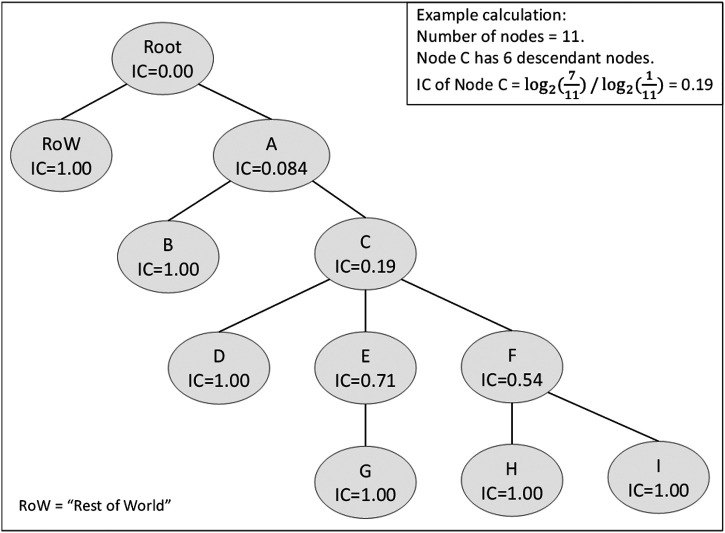
Example ontology IC calculation. The tree diagram depicts an example ontology with IC values calculated for each node. The RoW (Rest of World) node designates any entities not represented in ECO. An example of IC calculation for node C is shown in the top-right inset.

*K*_*w-agree*_ uses a weight matrix, *w*_*ij*_ *,* that contains the weight of the agreement between any pair of ECO terms. Here we use the information content (IC) of a pair’s lowest common ancestor as the weight for the Cohen’s *K*_*w-agree*_. (Seco et al., 2004). The IC calculation of each ECO term is based on the number of descendants that the ECO term has (Eq. 3; Figure 4). Then, the weight of agreement in *K*_*w-agree*_ for a pair of ECO terms is the IC value for their lowest common ancestor. The largest IC value, 1.0, is *w*_*max*_ in Eq. 2, and corresponds to nodes with no descendants (leaf nodes).

$$IC_{ont}=\frac{\log_{2}\left(\frac{NumDesc+1}{TotalNumNodes}\right)}{\log_{2}\left(\frac{1}{TotalNumNodes}\right)}$$ (3)

where *NumDesc* is the number of descendants of a node, and *TotalNumNodes* denotes the total number of nodes in the ontology. The IC measure hence assigns lower agreement weight to matches between non-specific terms than between very specific (e.g. leaf node) ones. It also lowers the agreement weight for mismatches between distantly related terms and between descendants of heavily populated branches. For consistency, we include a rest-of-world (RoW) term that designates any object not included in ECO. This node is connected to ECO via an additional root node (Figure 4) that operates as the interface between the ontology and the external world. This root node has an IC of 0.0, capturing the notion that pairing an annotation to an ECO term and one to the outside world (i.e. no annotation) is the most severe form of disagreement possible.

Curators can assign multiple ECO terms to a sentence or sentence pair. To properly compute KwIC, annotations must, therefore, be aligned. This multi-label alignment occurs as follows, considering each sentence in turn.

1. If neither curator annotated the sentence, count a RoW-RoW match.
2. If one curator annotated the sentence and the other did not, count a mismatch between the ECO term and RoW. Each ECO term in the annotated sentence is counted.
3. If both curators annotated the sentence, consider all combinations of the ECO pairings between the two sets of annotations for this sentence, and use the best IC value for each pairing to align the annotations and determine which pairings to count. If one curator has unmatched ECO terms, count these as a mismatch between each unmatched ECO term and RoW.

### Simulation of KwIC Values

A simulated annotation process was used to determine the expected range of KwIC values for the corpus. Parameter estimation was performed on corpus documents and simulation was performed on synthetic documents of 1,500 sentences for the computation of KwIC values, which were estimated based on independent 100 replicates for each experiment. A complete description of the parameter estimation and simulation procedures is available in Supplementary Material 5.

### Selection of the Examples of Usage for ECO

We developed Python scripts to process annotations and select candidate examples of sentences and sentence pairs for the OBO field “example of usage”. Candidates were selected from among those annotations in which two or three annotators chose the same ECO term for an annotation in that sentence. Examples containing more than one ECO annotation were permitted, although the example was proposed only for the ECO term with two or three annotators’ agreement. The script grouped these annotations by ECO terms and by the Confidence attribute values. Up to four examples were chosen for ECO terms and were subsequently vetted manually by ECO curators to choose illustrative examples of use for a variety of ECO terms.

## Results and Discussion

### Defining a Pipeline for Annotation of Evidence-Based Assertions

Before a curation task begins, it is necessary to define precisely the goal and scope of the effort. The primary aim of this work was to generate a corpus that captured ECO annotations of evidence statements supporting a specific set of types of assertions, providing annotations that were sufficiently defined to support text mining systems. Evidence statements supporting assertions can, in principle, be found in all sections of a scientific manuscript and span multiple paragraphs or sections. Previous work has shown that the largest concentration of evidence-based assertions are found in the Results or Results and Discussion section of the manuscript (Crangle et al., 2007; Islamaj Dogan et al., 2017b), and our preliminary review confirmed this observation. Although previous studies showed that figure and table captions are enriched in curatable information (Singhal et al., 2016), these typically detail evidence backing up assertions in the text, not in the captions themselves. Thus, we selected only Results or Results and Discussion sections for annotation.

Initially, we also constrained curation to individual sentences containing both mention of evidence and an assertion based on that evidence. To assess the effect of constraining annotation to this type of individual sentences on annotation coverage in the corpus, we randomly selected 12 documents from the 84 in the corpus, and we had an expert curator annotate all evidence statements supporting assertions without constraints (e.g. spanning multiple paragraphs or collating multiple sources of evidence). This resulted in 182 annotations for a total of 773 annotatable sentences (i.e. an annotation density of 23.54%). We then repeated the process, but annotating only self-contained sentences. Out of the 182 annotated unconstrained annotations, 94 were captured as self-contained sentence annotations (51.6%). During the preliminary review, observations from curators indicated that, for multiple assertions, evidence was often stated in the immediately preceding or succeeding sentence, so we assessed the impact of considering also consecutive sentence pairs for annotation. Using both individual sentences and sentence pairs raised coverage to 143/182 (78.6%; 18.50% annotation density) without dramatically increasing the complexity of the annotation effort, and we adopted this approach for the entire corpus.

Determining what constitutes an evidence-based assertion is a subjective process. The preliminary review indicated that curators often diverged in their interpretations of what constituted an evidence-based assertion. To capture, to some extent, this subjectivity, we introduced several qualifiers to the annotation. Curators were asked to assign a confidence value to their mapping of ECO term and text, and to determine the perceived forcefulness of the assertion. Furthermore, because some assertions are negative, we created an attribute that indicates if the annotation is negative or not. The annotation process, including annotation qualifiers, was condensed into a set of annotation guidelines and training materials that effectively instructed undergraduate curators on the goals and constraints of the annotation process, illustrating what to annotate (Figures 1 and 2) and what not to annotate (Table 1) with specific examples from the training documents.

**TABLE 1:** Example of sentences not appropriate for curation, with reason.

**No assertion—a statement of technique**
"We extracted 50 nucleotides directly upstream from each captured 5′-end, resulting in 1,451 sequences derived from the (delta)hrpL-FLAG sample and 1,472 sequences from the hrpL sample (overlapping sequences within a sample were merged) and used the sequences as input to MEME Pesquita et al. (2009).”
**No assertion—an observation of experimental output**
“We found that compared to that of wild type, toxR-lacZ expression was reduced in aphB mutants, while expression of aphB from a plasmid in this mutant restored toxR expression (Figure 4B) and ToxR production (Figure 4C).”
**No assertion—a statement of purpose**
“To confirm that *S. lividans* AdpA controls the expression of genes identified as differentially expressed in microarray experiments, six genes were studied in more detail by qRT-PCR.”
**No experimental evidence stated**
"Moreover, the inability to observe direct EspR-dependent regulation at some major EspR binding sites suggests that EspR has no or little effect on these genes in the conditions tested or that other regulators counter-balance the effect of increased EspR levels."
**Assertion not about one of the 6 categories**
"As expected, the ompF promoter activity (beta-galactosidase activity) decreased significantly in DeltaompR relative to WT grown at high medium osmolarity (0.5 M sorbitol); however, it showed almost no difference between WT and C-ompR, thereby confirming that the ompR mutation was nonpolar."
**Assertion too vague**
"Although the scan matched all annotated and new candidate hrp promoters identified in this study, the model did not match any other region in the genome that showed enrichment in the ChIP-Seq experiment (E_value_ cut-off = 0.001, 245 promoter candidates in total)."
**Evidence and assertion not in one sentence or two consecutive sentences**
Sentence #1: "The stacking energy profiles of *R. etli* and *E. coli* promoter regions were variable, but with a tendency to low negative values (low stability), nevertheless local minimum values were located around the -10 box."
Sentence #2: "In contrast, the stacking energy profiles of *R. etli* and *E. coli* coding regions were similar: Both showed more negative values that corresponded to great stability (Figures 2A,B)."
Sentence #3: "These results suggest that despite the variability of the nucleotide composition of the *R. etli* promoters, these regions possess thermodynamic and structural properties similar to the *E. coli* promoter regions."

### Corpus Overview

The ECO-CollecTF corpus contains the first textual annotations based upon the extensive, detailed evidence terms from ECO. It provides clear, self-contained evidence sentences and embraces a definition of evidence circumscribed to the context of an assertion. Thus, its goal and structure set it apart from previously developed corpora. Identifying occurrences of evidence backing assertions on biological entities is notably more difficult than tagging entities. The evidence annotations in the ECO-CollecTF corpus are therefore of significant import for the development of automated fact-based extraction methods, which must link asserted statements to their supporting evidence in order to provide experimental justification for the statements. On the other hand, the restriction of the annotation process to single or consecutive sentences generated a unique dataset of short text segments containing all the relevant elements of an evidence-based assertion. This provides a singular reference for text-mining, defining a significantly constrained task that can be leveraged for the training and testing of text-mining systems prior to their tackling the general problem of detecting and linking instances evidence and biological terms involved in assertions across a scientific manuscript.

A total of 84 annotated results sections make up the ECO-CollecTF corpus. Table 2 shows additional statistics about the corpus. As expected from the preliminary assessment, the inclusion of consecutive sentences enabled the inclusion of approximately 50% more annotations than those obtained when restricting to single self-contained sentences. The constraint of single or consecutive sentences resulted in a sentence annotation density of 14%, in line with but below that observed in the preliminary assessment, and likely resulting from the stricter adherence to the final annotation guidelines once they were formulated.

**TABLE 2:** ECO-CollecTF corpus statistics.

Number of unique documents	84
Number of annotated documents	282
Number of annotatable sentences	19,702
Number of annotations (total)	2,565
Number of consecutive sentence annotations	908
Number of sentences annotated (when split)	2,774
Average number of annotations per document	9.1
Number of unique ECO terms used	146

**FIGURE 5:**
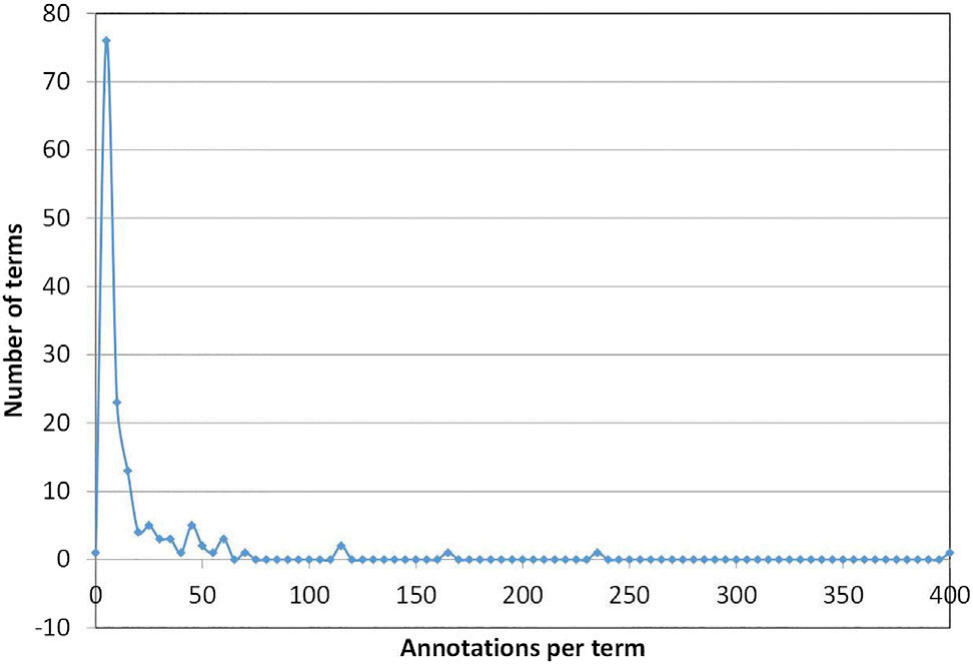
Annotation counts per ECO term.

As shown in Figure 5, the annotations in the ECO-CollecTF corpus were not uniformly distributed across the ECO terms. Of the 146 unique ECO terms used, over half of them (52.41%) are used only 1 to 5 times, and 15.86% between 6 and 10 times. Such term bias is not uncommon. For instance, in the CRAFT corpus (Bada et al., 2012), 3,657 of the 8,277 annotations for the cellular component sub-ontology of GO were for the term GO:0005623, “cell”. In the case of the ECO-CollecTF corpus, the observed bias results directly from the focus on articles about bacterial transcription factors, leading to a preponderance of experimental and computational techniques used in the study of transcriptional regulation, such as ECO:0000096 ‘electrophoretic mobility shift assay evidence’ (400 annotations) or ECO:0000028 ‘motif similarity evidence’ (112 annotations).

The ECO-CollecTF corpus includes the attributes to capture different subjective aspects of the annotation. “Assertion strength” captures the strength of an assertion supported by evidence, while “Confidence” attribute captures the quality of the mapping of the ontology entry to the text statement of evidence, as perceived by the curator. We found a clear bias toward “High” values for both subjective measures (Supplementary Material 6, Supplementary Material 7). This is likely due to the fact that, when faced with obliquely worded assertions and weak mappings to ECO terms, curators generally opted not to annotate the corresponding sentences. We also found that the distribution of “Confidence” was significantly skewed across ECO terms (Supplementary Material 7). This is partly due to the prevalence of some specific terms with well-defined text mappings (e.g. ECO:0000096 ‘electrophoretic mobility shift assay evidence’) and suggests that the definition of a significant fraction of terms in ECO could be modified to more closely align with their textual representation in journal articles.

### Inter-Annotator Agreement

The trustworthiness of biomedical corpora, when true negatives can be reliably estimated, is typically assessed with the Cohen’s K score (Artstein and Poesio, 2008). Here we implemented this approach by computing Cohen’s *Κ* score for all possible pairs of curators who annotated the same set of documents. As described in the Methods section, we then averaged the scores for each curator pair to obtain the overall Cohen’s *Κ* for the ECO-CollecTF corpus annotations, which is 0.69. In contrast to other corpora, this score is not extrapolated from a subset of documents annotated by multiple curators, but represents a *bona fide* estimate of overall corpus trustworthiness. The 0.69 score is comparable to reported *K* scores in annotation tasks of similar complexity (Herrero-Zazo et al., 2013; Jimeno et al., 2008; Véronis, 2001).

Cohen’s *K* and similar metrics measure binary agreement, in terms of whether curators agreed on annotating, or not, a given text unit. This approach works well for many biomedical corpora, since the focus is the identification of broadly defined entities in text. However, when annotating against an ontology, such as in the case of the ECO-CollecTF corpus, curators have the freedom to choose any of the terms in the ontology for a given annotation. In this situation, the annotation involves multiple classes to select from. Thus, the IAA metric should take into account the similarity of the ontology choices among the multiple possible selections.

Various techniques have been tried for applying ontology-based similarity (Pesquita et al., 2009) in order to derive weights for IAA scoring. These include common ancestor counts (Van Auken et al., 2014), child counts (Artstein and Poesio, 2008; Melamed and Resnik, 2000), or heuristics involving depth differences (Geertzen and Bunt, 2006). Here we build on the formal approach formulated by Seco *et al.* (Seco et al., 2004) to obtain weights based on the information content (IC) of each native ECO term in order to compute a weighted version of Cohen’s K, dubbed KwIC (Figure 4). This approach is similar in spirit to the one used in the BioCreative Gene Ontology task evaluation (Mao et al., 2014), and provides a principled, objective metric to measure the degree of similarity between two annotation choices from the ontology. The average KwIC score of the ECO-CollecTF corpus is 0.55. This value is, as expected, lower than the binary Cohen’s K score, since only annotations to terminal (leaf) nodes have full agreement weight (IC = 1).

**FIGURE 6:**
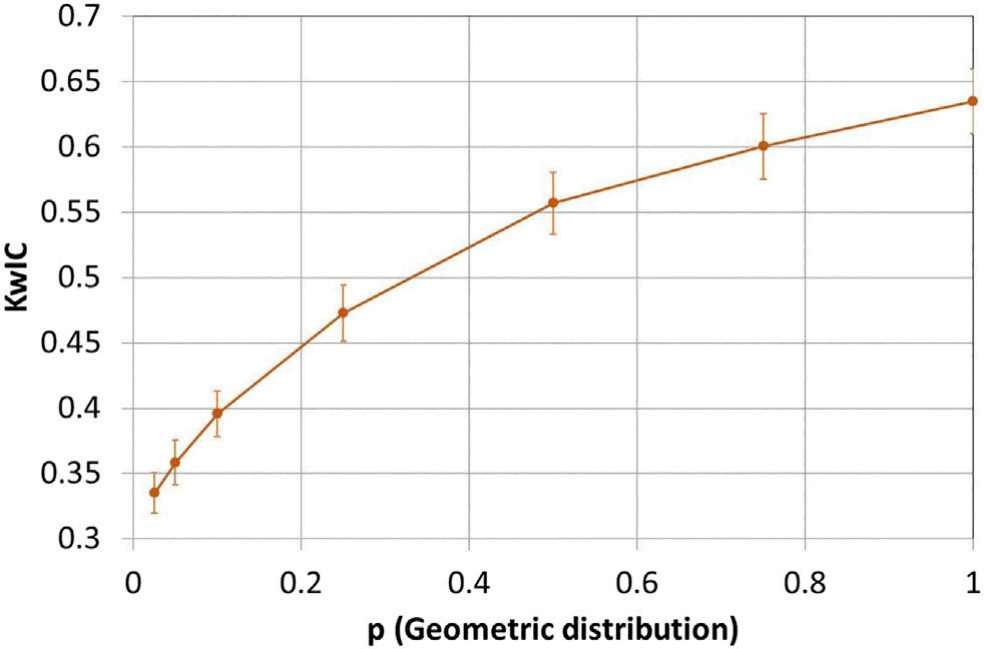
KwIC scores for different probabilities of success. *p* is probability of success, which provides the term distance between the two simulated curators. *p* = 1.0 is perfect agreement. KwIC averaged for 100 simulated annotated corpora at each *p*. Bars show standard deviation of the KwIC scores.

The transition to a weighted *K* index is appropriate in the context of multi-label annotation efforts (Cohen, 1968), but forgoes the context provided by comparisons with previous efforts to ascertain corpus trustworthiness. To address this, we sought to derive bounds and empirical estimates for the *KwIC* metric (Figure 6). Like *K*, *KwIC* has an upper bound of 1 (perfect agreement) and can achieve negative values when there is systematic disagreement (Cohen, 1960; Cohen, 1968). To obtain reliable estimates of *KwIC* in the context of our annotation effort, we estimated the annotation density, false positive and false negative rates of the corpus, and we used these estimated parameters to simulate the annotation process on 100 independently generated reference corpora. Assuming an annotation density of 14%, false positive rate of 2.4% and false negative rate of 14%, as estimated from the corpus, simulating perfect annotator agreement on corpora containing only ECO leaf nodes yields a *KwIC* value of 0.71 ± 0.02.

We next performed simulations using the corpus distribution of ECO terms, and varied the amount of disagreement between curators by imposing that one of the curators annotate a number of hops away from the other. The number of hops is randomly drawn from a geometric distribution with probability *p*. These simulations revealed that with perfect agreement (*p* = 1), the expected *KwIC* of our corpus is 0.63 ± 0.02. This is lower than the binary *K* value of 0.69 because a substantial fraction of annotations (∼45%) in the corpus are to non-leaf nodes, which by definition have IC weights smaller than 1. For low values of *p* (*p*→0), *KwIC* stabilizes at 0.0.32 ± 0.01. This is the result of the low density of annotations in the corpus (14%), which leads to a significant fraction of the tabulated results being counted as RoW-RoW agreements (IC = 1). The simulation experiments therefore provide adequate context to the value of *KwIC* = 0.55 observed for the ECO-CollecTF corpus, with a lower bound of 0.32 and an upper bound of 0.63. Taking into account this expected dynamic range for *KwIC*, the observed 0.55 value is roughly 75% of the maximum expected value (0.63; perfect agreement), indicating a substantial level of agreement between curators in the ECO-CollecTF corpus.

### Corpus Release

The ECO-CollecTF corpus follows the FAIR principles of being “findable”, “accessible”, “interoperable”, and “reusable”. The corpus is available in a public, permanent repository in two widely used formats, BRAT (Stenetorp et al., 2012) and BioC (Comeau et al., 2013), supporting interoperability and reusability. In addition, the ECO-CollecTF corpus is also available as an ECO OBO file that incorporates annotations for each term within custom JSON-formatted properties, facilitating accessibility and automated updating of the corpus. The corpus is linked to from the ECO website to enable findability. Together with the annotations, the parsed results and results/discussion sections for all journal articles are available in ASCII text files with one sentence per line in the permanent repository. All these sections are from journal articles published with the Creative Commons License allowing unrestricted, non-commercial use. The derivative versions of annotated documents are available under the original, open-access manuscript license. Annotations are available according to Creative Commons BY NC 4.0 license. The guidelines, training materials, examples, and code are also available with the same license.

In contrast with many other corpora, the ECO-CollecTF corpus supplies the original, individual annotations made by each member of the curation team, rather than a harmonized consensus. This makes it possible for users to fully reconstruct the original corpus, reproduce the IAA computations, subset it to include only annotations matching a particular attribute or, following the published guidelines, add their own annotations to the corpus. The inclusion of annotation attributes also has substantial bearing on reusability, since it provides important qualifiers (e.g. negative assertion) that can be leveraged by machine learning approaches to text-mining. This also applies to the annotation restriction to single and consecutive sentences, since it provides a unique dataset of well-defined, short text segments containing evidence-based assertions, and therefore defines a simpler, circumscribed text-mining task.

### Examplesof Usage of ECO Terms in Journal Articles

The restriction of annotation to single or consecutive sentences containing an evidence term involved in an assertion can be leveraged to enhance the ontology by including examples of use for its terms. This provides value to ontology users and curators, in the form of real examples of use that complement the ontology term definitions and assist curators in making informed decisions about the applicability of a given term in the different contexts. In addition, examples of use benefit the ontology developers by enabling them to understand how authors express ontology concepts in articles, helping them refine the ontology and providing external contrast when assessing changes in the ontology structure.

To generate adequate examples-of-use, we selected up to three annotations with unanimous curator agreement for each ECO term available in the ECO-CollecTF corpus. These annotations were manually reviewed by ECO curators, resulting in 63 usage examples attached to 45 terms added to ECO (SUPLXXX).

### Limitations and Target Audience

The ECO-CollecTF corpus is the first corpus dedicated to the annotation of evidence terms in scientific text using the reference ontology for evidence (ECO). As such, it introduces expert knowledge in defining what constitutes an instance of an evidence term in a scientific manuscript and provides a foundation for the development of corpora incorporating ontology-based annotation of evidence. As is often the case in seminal work, the ECO-CollecTF corpus has some limitations, which are outlined below:

- Size: the ECO-CollecTF is based on the curation of 84 documents with 2,565 annotations. While this is a modest number of documents, it is comparable in size to other seminal corpora, such as CRAFT (Bada et al., 2012), and to corpora focused on the topic subject matter of the ECO-CollecTF corpus (Bossy et al., 2012; Pyysalo et al., 2012).
- Annotation scope: the ECO-CollecTF corpus focuses on the annotation of ECO terms in scientific articles. The mapping of evidence terms is predicated on their support of an assertion involving a biological entity, but the entity itself and the relationship are not explicitly annotated in the corpus. The corpus, however, annotates self-contained sentences or sentence-pairs encompassing the evidence and the asserted entity, as well as the ontology of the corresponding biological entity. This provides a unique template to expand the corpus through entity annotation, as well as a well-defined benchmark for the development of text-mining tools for ontology-based entity and relationship tagging incorporating evidence.
- Article scope: the ECO-CollecTF corpus annotates only the Results (or Results and Discussion) sections of the manuscript. While this necessarily misses mentions of evidence in other sections of the document, such as figure legends, our results show that this approach captures a large fraction of evidence mentions used in assertions regarding biological entities, and provides a resource for focused text-mining initiatives.
- Thematic scope: the ECO-CollecTF corpus is restricted to the annotation of articles on bacterial transcriptional regulation. This was motivated by the expertize of the collaborating teams and devised as a means to focus the annotation effort. The procedures and results, however, are of general import to any targeted annotation efforts using ECO, and the corpus constitutes an important resource for ongoing efforts to annotate articles on transcriptional regulation (Lithgow-Serrano et al., 2019).
As the first corpus to directly annotate evidence in journal articles using the *de facto* standard ontology for evidence, the ECO-CollecTF corpus provides extensive guidance and a template for the annotation of evidence in biomedical corpora. Furthermore, its thorough assessment and validation of inter-annotator agreement using a metric that takes into account term ontological relationships also defines the expectations on corpus trustworthiness for similar initiatives seeking to annotate ontological terms at different levels of granularity. The corpus and its associated materials are therefore of interest to annotation teams wishing to incorporate evidence annotation to their curation process.

The ECO-CollecTF corpus comprises 2,565 annotations of ECO terms in sentences and sentence pairs that contain an explicit assertion about a biological entity. The annotations include quality and negation attributes, as well as the reference ontology for the asserted entity. As such, the corpus provides a unique benchmark for teams seeking to develop text-mining systems addressing not only the mapping of ECO terms in text, but their assessment as bona fide mentions of evidence through the identification of relevant biological entities and assertions in a constrained textual domain.

## Conclusion

In this effort, we defined evidence-based assertions, with attributes capturing the confidence in associating evidence text with an ECO term and the assessment of the forcefulness of the assertion, and developed guidelines for their curation. We created a corpus of 84 documents about TFBS in bacteria with 2,565 instances of evidence involved in assertions about different biological entities, with each document annotated by at least three curators. The corpus is the first, to our knowledge, with annotations of evidence terms using ECO, and 63 examples of use were selected from the annotations for inclusion in ECO. We also developed and characterized using simulations a novel IAA metric, *KwIC*, which extends Cohen’s *K* using information content based on the structure of ECO. In addition, all curator annotations are included in the corpus, allowing other researchers to generate a harmonized corpus or calculate the IAA using whatever methods they wish. The ECO-CollecTF corpus is a novel addition to the body of corpora available for the development of text mining systems and other applications.

## Data Availability

The datasets presented in this study can be found in online repositories. The names of the repository/repositories and accession number(s) can be found below: ECO-CollecTF v1.2 https://zenodo.org/record/4568935. DOI: 10.5281/zenodo.4568935.
