# Supplementary material for: ECO-CollecTF: A Corpus of Annotated Evidence-Based Assertions in Biomedical Manuscripts
Source: Front Res Metr Anal. 2021 Jul 13;6:674205. doi: 10.3389/frma.2021.674205 (PMC8313968; doi:10.3389/frma.2021.674205)
Supplement: Supplementary file 1 [file DataSheet7.PDF]

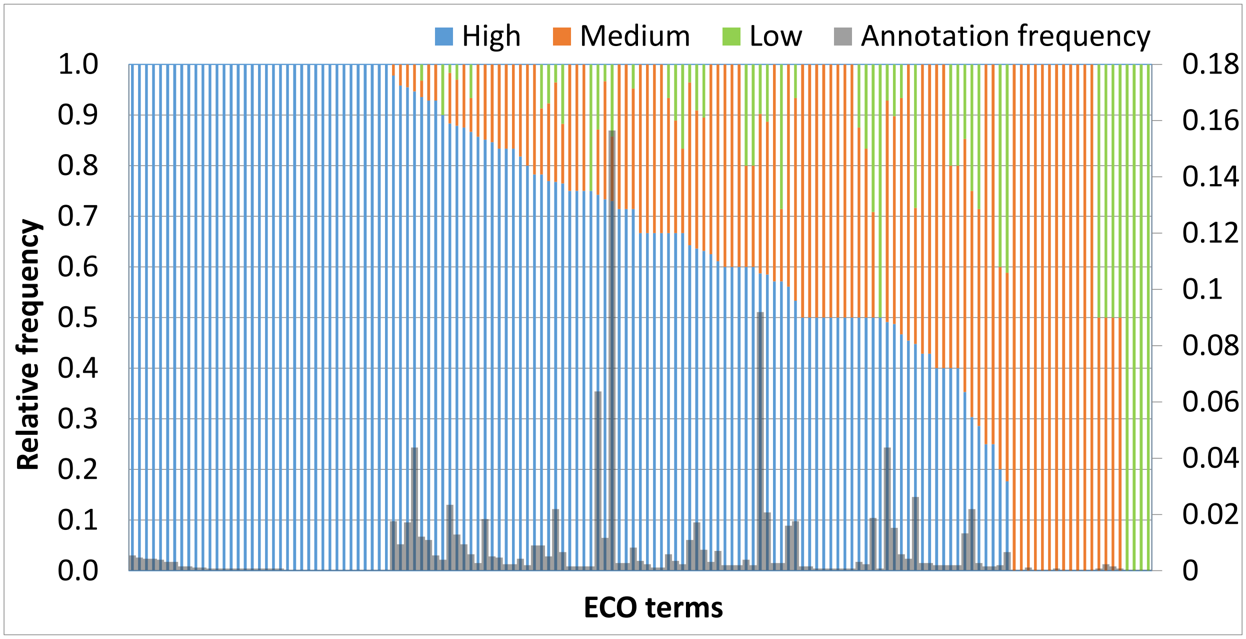

Relative frequencies of use of confidence values for ECO terms. Blue, red and green bars denote frequency of "High", "Medium", and "Low" Confidence assignments for each term (left vertical axis). Gray bars denote the overall frequency of use for each term. The "Confidence" attribute captures the quality of the mapping of the ontology entry to the text statement of evidence. The distribution is dominated by "High" confidence mappings (68.70%), with only 9.08% "Low" confidence annotations. The highest grey bar, corresponding to ECO:0000096 ('electrophoretic mobility shift assay evidence') contributes 73% of all annotations designated as "High".
