# Supplementary material for: ECO-CollecTF: A Corpus of Annotated Evidence-Based Assertions in Biomedical Manuscripts
Source: Front Res Metr Anal. 2021 Jul 13;6:674205. doi: 10.3389/frma.2021.674205 (PMC8313968; doi:10.3389/frma.2021.674205)

BRAT manual

Rules to remember

1. Create annotations for evidence + assertion within ONE sentence or a sentence PAIR.
2. Create an annotation for each ECO evidence that is used for an assertion (within a sentence or a sentence pair).
3. Create an annotation for each assertion that has ECO evidence in the sentence or pair.
4. There can be the situation where an annotation is made for a sentence pair (evidence in first sentence and assertion in second sentence) AND an annotation is made for the second sentence because it also has evidence + assertion within it.

General Instructions

BRAT annotations need to be done through the Chrome (or Chromium) browser. The Safari browser can also be used on a Mac. (See link at end for Powerpoint on using brat.) Firefox can also be used, but BRAT will complain about it (but still seems to work). BRAT has some quirks and little bugs. If it appears stuck, try resizing the window or moving to another document and back again to the one you are working on. Sometimes logging out and closing the browser and trying again will cause BRAT to stop misbehaving. Also, if you double click on an annotation to edit it and see that there are no drop down menus, first be sure you are logged in. If you are logged in, cancel the update window to dismiss it, then highlight any word as if you were going to annotate it, then cancel the new annotation. Subsequent annotations will have all the UI controls.

Perform the following steps to get into the BRAT server:

1. Bring up your browser and enter http://temu.bsc.es/ECO/brat/index.xhtml into the address bar.
2. On the first page, click OK.
3. Click cancel to dismiss the file browser box. Don't try to annotate before you log in. (You can pick your document first and then login but you won't be able to create or update annotations until you do.)
4. Hover your mouse over the blue bar at the top of the BRAT window on the right hand side and you will bring up a "login" link. Click that.
5. **Login** with the username and password you were given.

You are now ready to annotate. Begin by **selecting a collection and a document**.

1. Hover your mouse over the blue bar at the top of the window on the left hand side and you will see a choice for "Collection". Click that.
2. All training annotation documents are under ECO_Training. Double click on that directory.
3. Double click on the directory with your name.
4. You will see a list of documents. Double click on one to annotate. You can open a document with no annotations yet or one you've already worked on and keep annotating it.
5. When you open the document, you will see the contents of the file with each line on a separate line in brat (unless the sentence is very long, in which case it will wrap). Brat will

give line numbers in the left most column (line numbers include blank lines, so these are not sentence numbers).

6. See the section on how to create the annotation below.
7. When you are done annotating, hover your mouse over the blue bar on the right hand side to bring up the "logout" choice if you wish to exit your annotation session. Or, bring up the collection choice to get the file browser box to allow you to select another document if you wish to work on another article. The left and right arrows in the blue bar (left side) also allow you to step through your directory of documents.

To **create** an annotation:

1. Use the mouse to highlight one or more CONTIGUOUS words that are the ECO evidence source. Please do not highlight more than 5 or 6 words. If the evidence source is given in NON-contiguous words, please select the most important word or phrase in your judgement that indicates the kind of evidence.
2. Please try to not highlight extra spaces around the first and last word (as best as you can).
3. When you release the mouse button, a brat window will pop up.
4. If you don't like the text that you selected, pick cancel and there will be no annotation.
5. Otherwise, make sure ECO is selected as the entity type. **Please ignore any other types**.
6. Select the **ECO Confidence** from the drop down. This is your confidence regarding how well the words in the sentence map to the particular ECO term. How confident are you that the evidence is the term you've chosen based on the sentence words? (If the sentence says "EMSA" this would be a high confidence for ECO:0000096, but "shifted bands" would be low confidence.)
7. Select the **Assertion Strength** from the drop down. This is an assessment of how confidently/firmly the authors state their assertion. "Suggests" implies a lower strength than "conclude", for example. Assertion strength is not an indication of how closely aligned the author's statement is with (or maps to) any particular ontology concept or term. Assertion strength is not about the rate or manner of the results, so a statement about a "rapid increase" has no bearing on AssertionStrength. Something like "suggests a rapid increase" is a weaker strength than something like "shows a minimal increase".
8. Select the assertion **Category** from the drop down. Be sure the evidence given supports the assertion and is appropriate for that category.
9. If this is the first sentence of the **pair**, base your assertion on whatever is in the next sentence. Select Yes for the Next Sentence drop down.
10. If the evidence + assertion is in one sentence, leave Next Sentence blank.
11. If the assertion is a negative statement (like "we showed that X does not bind Y") choose Yes for **NegativeStatement**. You will probably have to scroll inside the menus' window to see that drop down. You can leave it blank for "No". (See at the end for an image of what to look for.)
12. Enter or search for the ECO ID.
13. To search for the ID click on the box to the right of the Ref: (it says "click here to search").
14. (Searching for ID continued:) This brings up another popup with the highlighted text ready to search. You can edit this text

15. (Searching for ID continued:) Click search brat_eco
16. (Searching for ID continued:) If the search finds something (should only take a couple of seconds), click the one you want and select OK.
17. (Searching for ID continued:) If there is nothing listed after a few seconds, try editing the search text and trying again. Or click cancel and search ECO's site directly.
18. If the ECO ID box is red, there is a problem. Re-enter the ID. If the box turns green, brat has accepted it.
19. OPTIONAL: enter a GO ID in the Notes at the bottom of the popup.
20. Click OK to create the annotation. The text will be re-displayed with the new annotation, which should be a light green color.

To **delete** an annotation:

1. Double click on the light green ECO icon containing the annotation you want to delete.
2. Brat will bring up a popup. At the bottom is a Delete button. Click this.
3. Brat will ask you to confirm the deletion. If you do, the annotation will be removed.

To **edit** an annotation:

1. Double click on the light green ECO icon containing the annotation you want to delete.
2. Brat will bring up a popup (same as shown when deleting).
3. Make your corrections.
4. Click the OK button at the bottom of the popup

NOTE: Normally brat works well. Occasionally it gets confused, for example, it may not respond properly when you try to double click on an annotation you've made. If it doesn't seem to be responding properly, try resizing the window. If that doesn't fix the problem, try logging out, closing your browser window, and re-entering (re-login, etc.) brat. If this doesn't fix the problem, please notify Terri.

The above contains a powerpoint demonstration using brat. Below is an image of the menu controls in the create and edit popup windows in brat. The NegativeStatement drop down menu is often hidden but can be obtained by scrolling using the vertical scroll bar on the right.

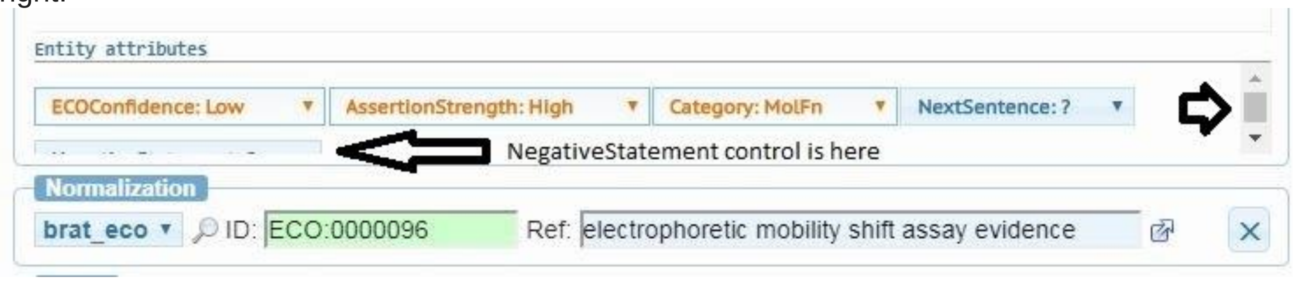

Supplement: Supplementary file 2 [file DataSheet2.PDF]
