# Supplementary material for: ECO-CollecTF: A Corpus of Annotated Evidence-Based Assertions in Biomedical Manuscripts
Source: Front Res Metr Anal. 2021 Jul 13;6:674205. doi: 10.3389/frma.2021.674205 (PMC8313968; doi:10.3389/frma.2021.674205)
Supplement: Supplementary file 3 [file DataSheet4.PDF]

Worked out Example for KwIC

a.

	**ECO:0000008**	**ECO:0000096**	**ECO:0005554**	**ECO:000555**
**ECO:0000008**	0.4604	0.02110	0.0005823	0.0005823
**ECO:0000096**	0.02110	1.0	0.0005823	0.0005823
**ECO:0005554**	0.0005823	0.0005823	0.7800	0.6057
**ECO:0005555**	0.0005823	0.0005823	0.6057	0.8257

ECO Term Pairs IC Values Matrix

Using Equation 3, calculate the IC for all ECO terms. Then, for each pair of ECO terms, find their lowest common subsumer. The IC value for the subsumer is the IC value for the pair that goes into the ECO Term Pairs IC Values Matrix. In this example, the lowest subsume for ECO:0005554 and ECO:0005555 has an IC value of 0.6057.

b.

	**Curator B**					
**Curator A**	**ECO:0000008**	**ECO:0000096**	**ECO:0005554**	**ECO:0005555**	**No**	**Total**
**ECO:0000008**	3	1	1	0	1	6
**ECO:0000096**	0	5	0	1	1	7
**ECO:0005554**	1	0	2	1	2	5
**ECO:0005555**	0	1	1	4	0	6
**No**	1	1	0	1	47	50
**Total**	5	8	4	7	51	75

Count Table for Annotator Agreement between Curators A and B

Construct the Count Table by aligning annotations between the two curators for each sentence as described in Methods, then count the number of ECO term pairs or the number of non-annotations (No). In this example, Curators A and B both annotated 3 of the same sentences with ECO:0000008. For another sentence, Curator A chose ECO:0000008 while Curator B chose ECO:0000096.

c.

	**Curator B**					
**Curator A**	**ECO:0000008**	**ECO:0000096**	**ECO:0005554**	**ECO:0005555**	**No**	**Total**
**ECO:0000008**	0.04	0.0133	0.0133	0	0.0133	0.08
**ECO:0000096**	0	0.0667	0	0.0133	0.0133	0.0933
**ECO:0005554**	0.0133	0	0.0267	0.0133	0.0267	0.08
**ECO:0005555**	0	0.0133	0.0133	0.05480	0	0.08
**No**	0.0133	0.0133	0	0.01370	0.6267	0.667
**Total**	0.0067	0.1067	0.0533	0.0933	0.68	1.0

Frequency Table (Rounded)

Construct the Frequency Table by dividing the Count Table values by the total number of annotations (here, 75).

d.

Curator A	Curator B					
	**ECO:0000008**	**ECO:0000096**	**ECO:0005554**	**ECO:0005555**	**No**	**Total**
**ECO:0000008**	0.0184	2.813×10^{-4}	7.764×10^{-6}	0	0	0.0187
**ECO:0000096**	0	0.0667	0	7.764×10^{-6}	0	0.0667
**ECO:0005554**	7.764×10^{-6}	0	0.0208	0.00808	0	0.0289
**ECO:0005555**	0	7.764×10^{-6}	0.00808	0.0440	0	0.0521
**No**	0	0	0	0	0.6267	0.6267
**Total**	0.0184	0.0670	0.0289	0.0521	0.6267	0.7931

Weighted Frequency Table

Construct the Weighted Frequency Table by multiplying each cell in the Frequency Table by the appropriate ECO Term Pairs IC value (Step a). For example, the cell [ECO:0000008, ECO:0000008] is 0.04 x 0.4604 = 0.0184.

e.

Curator A	Curator B				
	**ECO:0000008**	**ECO:0000096**	**ECO:0005554**	**ECO:0005555**	**No**
**ECO:0000008**	0.005333	0.008533	0.004267	0.007467	0.05544
**ECO:0000096**	0.006222	0.009956	0.004978	0.008711	0.06347
**ECO:0005554**	0.005333	0.008533	0.004267	0.007467	0.05440
**ECO:0005555**	0.005333	0.008533	0.004267	0.007467	0.05440
**No**	0.04444	0.07111	0.03556	0.06222	0.4533

Unweighted P_e Table

Construct the Unweighted P_e Table by multiplying the marginal frequencies in the Frequency Table (Step c) for each row and column. For example, the cell [ECO:0000008, ECO:0000008] = 0.08 x 0.0067 = 0.005333 (unrounded value).

f.

Curator A	Curator B					
	**ECO:0000008**	**ECO:0000096**	**ECO:0005554**	**ECO:0005555**	**No**	**Total**
**ECO:0000008**	0.002455	1.800×10^{-4}	2.484×10^{-6}	4.349×10^{-6}	0	0.002642
**ECO:0000096**	1.312×10^{-4}	0.009956	2.899×10^{-6}	5.072×10^{-6}	0	0.01009
**ECO:0005554**	3.106×10^{-6}	4.969×10^{-6}	0.003328	0.004523	0	0.007859
**ECO:0005555**	3.106×10^{-6}	4.969×10^{-6}	0.002584	0.006165	0	0.008758
**No**	0	0	0	0	0.4533	0.4533
**Total**	0.002593	0.01015	0.005918	0.01070	0.4533	0.4829

Weighted P_e Table

Construct the Weighted Pe Table by multiplying each cell in the Unweighted P_e Table (Step e) by the appropriate ECO Term Pairs IC value (Step a).

g.

P_o = Sum of all cells in the Weighted Frequency Table (Step d) = 0.7931

P_e = Sum of all cells in the Weighted Pe Table (Step f) = 0.4829

Kwic = (0.7931 – 0.4829) / (1.0 – 0.4829) = 0.5999
