# Supplementary material for: ECO-CollecTF: A Corpus of Annotated Evidence-Based Assertions in Biomedical Manuscripts
Source: Front Res Metr Anal. 2021 Jul 13;6:674205. doi: 10.3389/frma.2021.674205 (PMC8313968; doi:10.3389/frma.2021.674205)
Supplement: Supplementary file 4 [file DataSheet6.PDF]

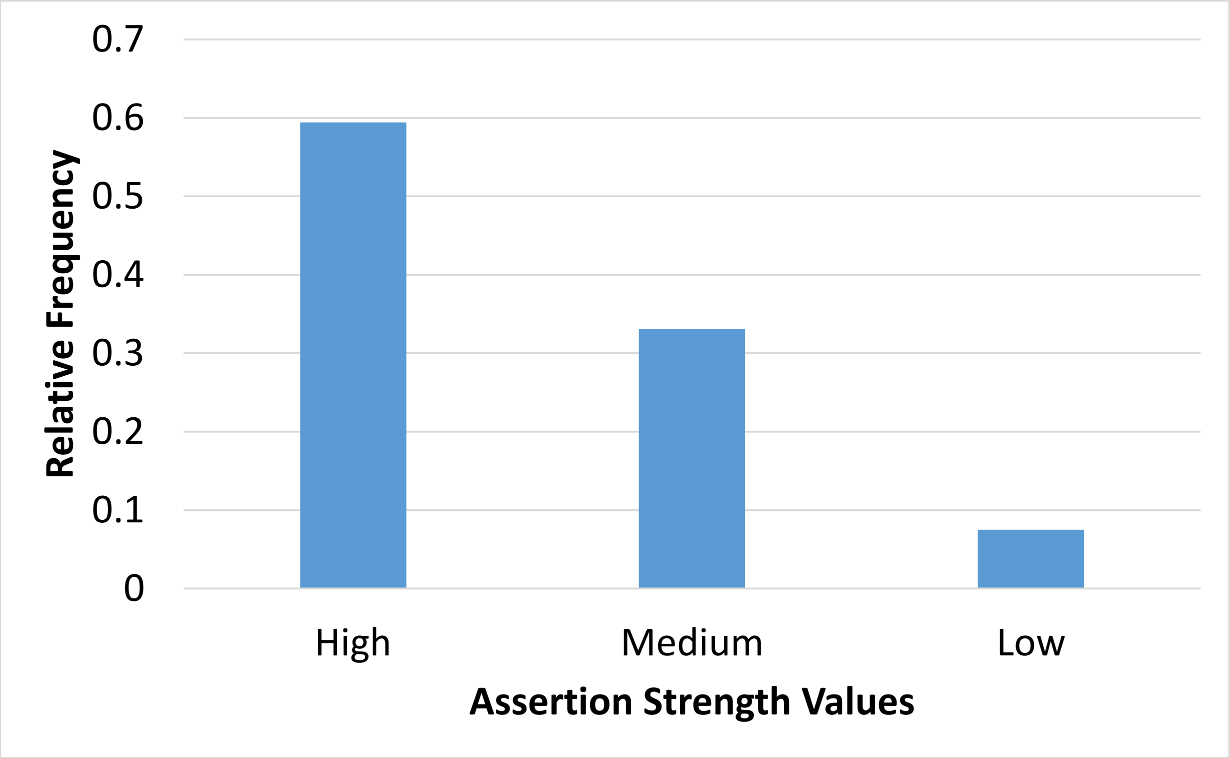

Relative frequencies of occurrences of “Assertion Strength” values in annotations. “Assertion Strength” captures the strength of the assertion as perceived by the curator. “Low” accounts for a very small percentage (7.1%), of the total number of annotations, whereas almost 60% of the annotations are considered to have “High” assertion strength.
