# Supplementary material for: ECO-CollecTF: A Corpus of Annotated Evidence-Based Assertions in Biomedical Manuscripts
Source: Front Res Metr Anal. 2021 Jul 13;6:674205. doi: 10.3389/frma.2021.674205 (PMC8313968; doi:10.3389/frma.2021.674205)
Supplement: Supplementary file 5 [file DataSheet3.PDF]

Worked out Example for Cohen's *K*

a.

		**Curator B**		
		Yes	**No**	**Total**
**Curator A**	**Yes**	8	2	10
	**No**	3	22	25
	**Total**	11	24	35

Count Table for Annotator Agreement between Curators A and B

Construct the Count Table for curator agreement between curators A and B by comparing annotations made by the two curators. Yes=Annotated. No=Not Annotated. In this example, curators A and B annotated 8 of the same sentences. Curator A annotated 2 sentences that Curator B did not, and Curator B annotated 3 sentences that Curator A did not. There were 22 remaining sentences out of the 35 total that neither curator annotated.

b.

		**Curator B**		
		**Yes**	**No**	**Total**
**Curator A**	**Yes**	0.2286	0.05714	0.2857
	**No**	0.08571	0.6286	0.7143
	**Total**	0.3143	0.6857	1.0

Frequency Table for Annotator Agreement between Curators A and B

Construct the Frequency Table for curator agreement between curators A and B by dividing all values in the count table by the total number of annotations (which is 35).

c.

Calculate the observed (P_o) and expected (P_e) proportion of agreement and Cohen's *K* from the Frequency Table.

$P_o = 0.2286 + 0.6286 = 0.8571$

$P_e = (0.2857*0.3143) + (0.7143*0.6857) = 0.5796$

$K = (0.8571-0.5796)/(1.0-0.5796) = 0.6602$
