# Supplementary material for: ECO-CollecTF: A Corpus of Annotated Evidence-Based Assertions in Biomedical Manuscripts
Source: Front Res Metr Anal. 2021 Jul 13;6:674205. doi: 10.3389/frma.2021.674205 (PMC8313968; doi:10.3389/frma.2021.674205)
Supplement: Supplementary file 6 [file DataSheet1.PDF]

# How to Annotate

## ECO terms

The Evidence and Conclusion Ontology (ECO) was developed to systematize the concepts underlying the use of evidence in scientific articles. *Evidence*, per se, is only part of the equation when scientists make inferences about the world. The other part is the *assertion method*; that is, whether the call is made by an automated system or by researchers taking a hard look at the data they gathered in the experiment. So ECO is divided in two main sub-ontologies: evidence terms and assertion methods (of which there are for now only two). For instance, the evidence term "*ECO:0000096 electrophoretic mobility shift assay evidence*" describes a type of experiment, while the derived term "*ECO:0001807 electrophoretic mobility shift assay evidence used in manual assertion*" describes a possible use of the experiment.

To simplify the process of ECO annotation, we will *only* use native evidence terms (like ECO:0000096), which define the types of evidence.

## Reading a manuscript

For ECO annotation, there are two main sections of the paper that are typically relevant:

- **Materials and Methods** section (aka. *Data and Methods*, *Methods*)
  - This is the section in which experimental methods are described. **We will not be using this section for the purposes of annotation**, but we can refer to it when trying to figure out which specific ECO term is being referred to in a sentence within the *Results* section.
- **Results section** (aka. *Results and Discussion*)
  - This is the section of the manuscript in which the experimental methods are mentioned in the context of an assertion. That is, the manuscript authors will often make some claim about some entity (e.g. a protein is observed to bind DNA) based on some evidence. **This is the section of manuscripts that we will be annotating**.
  - The authors are likely to make a claim regarding the nature of the assertion (whether they made a call based on the observed data from some experiment [a manual assertion] or they are using directly the output of some method as *bona fide* evidence [an automatic assertion]). These are the instances of ECO terms that we wish to capture.

## Performing the annotation

ECO annotation will be performed using the dedicated BRAT server. Once we log in and select one of the papers assigned to us to annotate, we will be able to tag specific sections of text to ECO terms (identifiers). Refer to the BRAT manual page for step-by-step instructions on

performing annotations with BRAT. All the manuscripts that we will be annotating are freely accessible through PubMedCentral (PMC). You can download the text or PDF version of the manuscript by looking it up with its PMID on PubMedCentral or PubMed.

## What to annotate

We will only annotate ECO terms in sentences that comply with the following requirements:

- Based *on the sentence* ***alone or in 2 consecutive sentences (a pair)***, we can determine that an evidence term (an ECO term) is being mentioned.
- Within the *same sentence or pair*, some ***assertion*** is made *based on the evidence* term. That is, the authors make a claim about some entity and it is explicitly statement or clearly implied that the claim is made based on the evidence.

In other words, the evidence description and its use to make a claim about some entity must be clearly stated and self-contained within the sentence (or within an adjacent pair of sentences).

Here is an example from PMID:17261178 with ECO term ECO:0000226 (chromatin immunoprecipitation evidence ):

```
The ChIP experiments also confirmed FNR-binding to the promoter regions of
the most highly FNR-activated transcript (NMB1205) and ompU.
```

The above sentence has "ChIP experiments" (a type of **evidence**) being used to **assert** ("confirmed") the binding of FNR to specific DNA regions.

A second example is from PMID:17892462 and ECO term ECO:0001810 (DNase footprinting evidence):

```
Previous in vitro DNase I protection analysis with PhylE identified a SlyA
footprint from -70 to -30 relative to the hlyE transcription start site,
reflecting the presence of two separate palindromic SlyA-recognition
sequences in this upstream region, these being SlyA1a (-61TTATCATATTA-50) and
SlyA1b (-50ATAGAAATAAG-39) (consensus-matching bases in bold)(Wyborn et al.,
2004b).
```

The text talks about *"DNase I protection analysis"* (a type of **evidence**) being used to **assert** ("*identified*") that there is a footprint at a specific location with particular characteristics (the palindromic recognition sequences) (sequence features).

Some sentences contain multiple evidence types. For example (from PMID:24694298):

```
S. lividans AdpA directly regulates at least the six AdpA-dependent genes
listed above and identified by microarrays and qRT-PCR analysis.
```

The above sentence has **evidence** "microarrays" (ECO:0000058, expression microarray evidence) and "qRT-PCR analysis" (ECO:0001566, quantitative reverse transcription polymerase chain reaction evidence ). It explicitly **asserts** that AdpA regulates other genes.

Evidence words in the sentence may be clearly related to a technique, but other evidence statements may be equally specific but not stated with explicit technique words. Consider this next sentence from PMID:24694298:

```
BLAST analysis revealed that the S. griseus AdpA DNA-binding domain is
conserved in S. coelicolor and S. lividans AdpAs (data not shown) suggesting
that all three species share the same AdpA-binding consensus sequence.
```

This sentence contains two types of evidence and two assertions. "BLAST analysis" ECO:0000206, is the evidence for the conserved assertion (the evidence "revealed" what is being asserted). Then the three species are used as sequence orthology evidence (ECO:0000201) to make an assertion about the consensus sequence (asserted with the word "suggesting").

## Objects of assertion

In the last section we determined that we would only consider only sentences that contained an assertion (based on the evidence) regarding some type of entity. To circumscribe the annotation effort, here we will only consider assertions on the following objects:

- Gene products (proteins, RNAs)
    - The entity of the assertion (what the assertion is about) is the product of a gene. Usually these are the subjects of an action or location (protein X binds DNA region Y -- X is the subject and is the gene product for which the assertion is made (the binding)). For reference, we will use the Gene Ontology (which describes aspects of gene products). In an annotation we will further specify whether the assertion corresponds to one of the following categories:
        - The **molecular activity** of the gene product (Gene Ontology Molecular Function)
            - Example (from PMID:24694298): "*These EMSA experiments demonstrated that S. lividans AdpA directly binds to five intergenic regions and confirmed the in silico prediction presented in Table 2.* "

```
Using EMSA assays, the authors state that the AphA protein binds a specific
regions of DNA, hence targeting a specific DNA sequence in the DNA. This is
captured by the GO molecular function term GO:0043565 (sequence-specific DNA
binding).
```

- 
    - 
        - The **biological/biochemical process** a gene product participates in (Gene Ontology Biological Process)
            - Example (from PMID:26793169): *Our microarray analysis showed that MafR influences positively the expression of numerous PTS genes*

```
Based on micro-array data (ECO:0000104), the authors state that MafR
positively regulates the expression of some genes (GO:0010628 - positive
regulation of gene expression), which is a biological process.
```

        - The **location** of the gene product inside or outside the cell ([Gene Ontology Cellular Component]())
            - Example (from PMID:24490131) which is a sentence pair: *The endogenous protein expression and localization for WalR and WalK was also checked using confocal immunofluorescence microscopy. WalR could be localized to the cytoplasm of B. anthracis but WalK could not be detected once again (data not shown).*

```
The first sentence provides the evidence, ECO:0005600, immunofluorescence
confocal microscopy evidence, as well as the purpose (expression and
localization). The second sentence asserts that WalR could be localized to
the cytoplasm. The two sentences together give the evidence type and tie the
evidence to the assertion. Because WalK couldn't be detected, no assertion is
made -- lack of detection is a readout. The assertion that is made is only
for cellular location.
```

        - GO category disambiguation:
            - Distinguishing between the three main sub-ontologies of the Gene Ontology can be sometimes tricky, especially between *molecular function* and *biological process*.
            - Read the guidelines on the links above for reference. In general, think about *molecular function* as an *intrinsic property* of a gene product (e.g. a peptidase cleaves peptide chains) and as *biological process* as a *role* of that function in a larger *context* (e.g. a peptidase can participate in "targeted protein degradation").
            - A gene product can have multiple functions (e.g. a peptidase will typically bind polypeptides) and participate in multiple processes (e.g. a peptidase may participate in the cellular response to antibiotics by degrading them). If multiple characteristics of the gene product are asserted in the sentence (or sentence pair) and the characteristics are shown by the evidence, annotate all different assertions.
            - As a rule of thumb, molecular function terms tend to be associated with in vitro techniques, biological process ones with in vivo methods terms, and cellular component ones with imaging techniques.
            - Common molecular functions: binding to DNA or to another protein, enzyme activity
            - Common and example biological processes: regulation of gene expression, stress responses, biosynthesis of molecules, regulation of metabolic processes, regulation of luminescence, regulation of copper ion homeostasis, cell adhesion
            - **NOTE you do NOT have to determine the actual GO term/identifier, only the category**.
- Biological sequences (Sequence Features)
    - The object of the assertion is a DNA, RNA or protein sequence. The assertion will typically be on a feature (e.g. a gene, a promoter element, binding site, the

chromosomal origin of replication...). Here for reference we will use the Sequence Ontology, and predominantly the sequence_feature class.

    - Example (from PMID:26793169): *'Sequence analysis of such a region revealed the existence of a putative promoter (Pma) (Figure 1), which shows a 4/6 match at the –10 hexamer (5'-TATTCT-3') and a 5/6 match at the –35 hexamer'*

This sentence uses a type of sequence analysis ('ECO:0000028 - motif similarity evidence') to assert that there is a promoter element upstream of gene *Pma*.

- Phenotypes and traits
  - The object of the assertion is a phenotype (e.g. the loss/gain of the ability grow on acetate as a carbon source) or a phenotypic trait (e.g. the loss/gain of the ability to develop wings, or fly, in birds). For bacteria, we'll use the Ontology of Microbial Phenotypes as our main reference, although phenotypes can extend to aspects not considered there.
  - Important: if the sentence discusses a specific gene or protein, we have a rule that it must be annotated using one of the Gene Product categories -- Molecular Function, Biological Process (or Cellular Location) categories -- not Phenotype. And it may be the case that the statement is then only a readout and not an assertion.
  - Correct Phenotype Example: (from PMID:25170934): *"We first confirmed that cells bearing the tagged protein retained the ability to stimulate the hypersensitive response in a plant assay (Figure 1B)..."*

Here, the authors show that their tagged cells are able to create a hypersensitive immune response in plants, a phenotypic reaction (ECO:0000059).

NOTE: no specific protein or gene is discussed in the sentence portion above; thus, Phenotype is an appropriate category.

- Taxonomic and phylogenetic objects
  - The object of the assertion is a cladistic assignment of some sort (e.g. the bacterium under study belongs to the Nitrosomonadales order) or a phylogeny-based statement about a gene (e.g. stating based on sequence similarity that two genes are orthologs). A reference for taxonomy is the NCBI Taxonomy server.
    - Example (from PMID:22233679): "*Phylogenetic analysis of the amino acid sequences spanning from region 2 to region 4 of the RpoQ, RpoS, and RpoD homologues among two species of the Vibrionaceae (V. fischeri and A. salmonicida) and Escherichia coli indicates that RpoQ is a protein that is distinct from RpoS (Fig. 2B).*"

Here the authors state that RpoQ is not related to RpoS, based on phylogenetic evidence (ECO:0000080)

        - Example (from PMID:22984476): "*Three clades I, II, and III were arbitrarily identified for the nine Vibrio species on the basis of phylogenetic tree."*

```
Here the authors state that they identified, and arbitrarily named, three
distinct clades (a phylogenetic grouping) based on phylogenetic evidence
(ECO:0000080)
```

## What NOT to annotate

There are several recurring cases of sentences that we will *not* annotate.

- Sentences lacking explicit mention of the **evidence**. This encompasses several common cases:
    - anaphoric (e.g. "the experiments above show") and cataphoric ("as seen below, this demonstrates") references
        - Example (from PMID:26793169): '*Additional experiments confirmed that promoter Pma drives transcription of the gfp gene in plasmid pAST-Pma'*

```
'Additional experiments' makes reference to some evidence, but this is not
detailed within the scope of the sentence.
```

    - implicit evidence, where we have to infer (from details in the assertion) that evidence is used
        - Example (from PMID:22984476): '*The results showed that His-AphA was able to bind to the DNA fragment of qrr4 rather than qrr2-3*'

Here we *could* assume that some sort of DNA-binding evidence is used, but we will not annotate such *implicit evidence*.

- Sentences that are a "wrap up" of several previous sentences. For example, a paragraph may have 3 sentences that each present observations from one or more experiments. Then the last sentence may say, "These results show..." Here "These results" refer to all 3 of the previous sentences. Since we are currently only interested in adjacent sentence pairs, we cannot annotate these "wrap up" sentences.  Note: there are some exceptions to this general rule. If the wrap up sentence repeats the evidence, it can be annotated. Also, in some situations, part of the wrap up sentence might have an assertion that clearly only relies on evidence in the previous sentence. In this case, it can also be annotated. But again, if the assertion really does require evidence from more than the previous sentence, it should not be annotated.
- Sentences that have an intervening non-evidence statement. Sometimes a paragraph will have a sentence that describes the experiment to perform (the evidence), followed by another sentence that provides other information (such as background data). Then the assertion is given in the third sentence in the sequence. Because the evidence and the assertion are not adjacent, we won't create an annotation.

- Sentences lacking an explicit **assertion**. These sentences give a statement of what was observed -- a readout -- and require the reader to make an inference to complete the assertion. We are not annotating readouts or inferred assertions.
    - Example (from PMID:21966533): "*As further determined by DNase I footprinting (Fig. 3d), the purified His-RovA protected two distinct regions upstream of rovA against DNase I digestion in a dose-dependent manner.* "
    - Example (from PMID:21966533): "*Under conditions I and III, there was no significant difference in the rovA promoter activities in the WT and DeltaphoP strains.* "
    - Example (from PMID:19236707): "*For instance, yibD was upregulated ~69-fold in the preAB mutant with pBAD-preA compared to the ~2-fold seen in PreB+ backgrounds, while mdaB was upregulated ~7-fold versus 2-fold in the PreB+ background.*"
    - Example (from PMID:21345178): "*The ompX gene was discarded by SAM in the microarray assay (which could be attributed to the fact that the repeatability of the 8 replicated data points of this gene were unacceptable by SAM), although it gave a more than 2-fold mean change of expression between WT and DeltaompR.* "
    - Example sentence pair (from PMID:21345178): "*The mRNA levels of each of ompC, F, and X were compared between DeltaompR and WT at 0.5 M sorbitol using real-time RT-PCR (Figure 2a). The results showed that the mRNA level of ompC, F, and X decreased significantly in DeltaompR relative to WT.* "

Note that the first sentence has clear techniques (mutant phenotype and real-time RT-PCR). But the second sentence only states what was observed and does **not** contain an actual assertion -- namely that OmpR regulates the expression of OmpC,F, and X. Hence, no annotation.

- Sentences lacking an explicit assertion regarding one of the target entities
    - Example (from PMID:26793169): '*By RT-PCR experiments (Figure 2), we analyzed the expression of the mafR gene in enterococcal V583 cells.*'

Here the evidence is clearly identified (RT-PCR), and a specific target object is mentioned (*mafR* gene), but no clear assertion is made. The sentence just indicate that research was done, but does not outline any results or inferences thereupon.

## Other Information about an Annotation

### Sentence Pair

The annotation indicates if it is for a sentence pair or not.

### Negative Assertion

The annotation indicates if the assertion is "negative" or not.

- Example of a negative assertion from PMID:24086521: "*Interestingly, primers spanning the 42 bp intergenic region between the putative znuB and a hypothetical gene, PA5502, did not give a product, suggesting that gene PA5502 is not part of the zur operon, despite its proximal location to the zur-znuC-znuB operon.* "
- Example of a negative assertion from PMID:21799779: "*However, no binding of CopR to the promoter region of cg0414 was observed in EMSAs (see Fig. S1), indicating that cg0414 is not a direct target gene of CopR.* "

## Annotation confidence

Annotation is a subjective process. In the process of ECO annotation undertaken here, we will distinguish between two different types of confidence: (1) the confidence on the mention of a specific ECO term and (2) the strength of the assertion made by the authors.

- ECO term confidence (Low/Medium/High)
  - This captures your belief that a specific ECO term is referred to in the sentence. In some cases, you'll be quite certain (e.g. when a specific technique matching an ECO term is mentioned explicitly), and in other cases you may have to infer from some clues in the sentence what the appropriate ECO term is. Below are examples of low, medium and high ECO term confidence.
  - High ECO term confidence example (PMID 22984476): *The primer extension experiments detected a single transcription start site located at 200 bp upstream of aphA.*

```
Here the evidence is determined from "primer extension experiments" to point
to ECO:0001819, primer extension assay evidence. (Aside: the category is
Sequence Feature.)
```

  - High ECO term confidence example (PMID:23209661): "*By EMSA, we found that PsrA directly binds its promoter region (Fig. S2A).* ".

```
Here the evidence is determined from "EMSA" and points to ECO:0000096,
electrophoretic mobility shift assay evidence. (Aside: the category is
Molecular Function.)
```

  - Medium ECO term confidence example (PMID:19236707): *"The beta-galactosidase activity assays (Figure 1) showed an evident upregulation of nrdAB, nrdHIEF and nrdDG expression in the absence of NrdR when* ***compared to the wild-type strains****, indicating that NrdR acts as a repressor of all three ribonucleotide reductases in S. Typhimurium LT2.* "

```
Here the evidence is determined from several sources. "Compared to wild-type
strains" does not clearly state the fact there was a mutant involved, so this
is ECO:0000015, mutant phenotype evidence, only at the medium level (given
that the phrase "compared to" is present it is more than low).
"Upregulated...expression" points to ECO:0000008, expression pattern
```

```
evidence, at a high level. It is not ECO:0000009 because no explicit mention
that transcripts were measured is stated. "Beta-galactosidase activity
assays" clearly points to ECO:0001802 at the high level.
```

- Low ECO term confidence example (PMID 22984476): *The presence of AphA box-like sequences within the promoter-proximal DNA regions of* aphA, qrr4*, and opaR in* V. parahaemolyticus *(Table 2) indicated that these QS regulators-encoding genes might be the direct AphA targets in V. parahaemolyticus, which were further validated by the following gene regulation experiments.*

```
Here there are two relevant ECOs. The evidence is determined from "box-like
sequences"  to point to ECO:0000028, motif similarity evidence. In
addition,  because these genes are called "AphA targets" the evidence is from
their responding similarly in expression experiments  .
```

## Assertion Strength

- Assertion strength (Low/Medium/High)
  - This captures your assessment on the strength of the assertion being made. That is, how strongly the authors state their claim. The assessment stems from the use of verbs and auxiliaries in the sentence. For instance, a very strong assertion could be of the form: "Based on the ... and ... experiments, we conclude that gene X is actively involved in process Y". A weaker assertion could be of the form: "The results of the ... assay suggest that protein X may be have Y activity". Below are examples of low, medium and high assertion strength.
  - Note that assertion strength is **NOT** the same as the manner of the experimental results. For example, an experiment could show "strong induction" or "rapid growth" but the assertion might be phrased along the lines of "rapid growth may indicate". "May indicate" is not a strong assertion (could be medium or low depending on the sentence).
  - Assertion strength does also **NOT** measure the alignment between the asserted entity in the sentence and any of the ontologies targeted for annotation. That is, assertion strength does not measure how well the statement "Based on xyz evidence, protein AbcX protects this segment of DNA" maps to the GO molecular function term GO:0043565 "sequence-specific DNA binding" that it is implicitly talking about. In other words, it works as well as "Based on xyz evidence, protein AbcX binds this DNA in a sequence-specific manner". As a curator you should only determine whether there is an assertion about molecular function therein, not how well-aligned it is with any ontology entry.
  - High assertion strength example (PMID:24086521) sentence pair: *Recombinant Strep-tagged ZurPA (rZur) was expressed and purified from E. coli strain BL21(DE3) (prZur) and used in EMSA experiments*. *P. aeruginosa rZur bound specifically to the 198 bp fragment which contains the znuA promoter in intergenic region spanning between znuA and zur (Figure 6).*

The ECO term confidence would be high (ECO:0000096, electrophoretic mobility shift assay evidence). The assertion is also high. In the case of binding experiments, it is enough to state that X binds Y or that X bound Y.

- Medium assertion strength example (PMID:23823757): "*Elevated CAT activities were detected in both the wild-type FW213 and DeltafimR harboring the pfim(109 b)-cat fusion, suggesting that the sequence between -151 and -109 contains a negative regulatory element*."

While the ECO evidence is high confidence (ECO:0000015, mutant phenotype evidence and ECO:0000049, reporter gene assay evidence), the word "suggesting" puts the assertion strength at medium.

## Description of previous research

Sometimes, authors will describe previous research in some detail, including the methods and inferences made by other authors. For instance, in a manuscript we may find a sentence like: "Using yeast-to-hybrid Chen *et al.* were able to identify CrmR as a binding partner of HrpX". This type of sentence satisfies all the criteria we are looking for (evidence is clearly stated and used to make a clear assertion about an entity), and hence we will annotate it in the same way as we do for sentences in which the authors report their own findings.

## Performing the annotation

Before starting the annotation, you should carefully read the *Materials and Methods* and *Results* sections to understand the overall context of the manuscript and identify what appear to be *bona fide* mentions of ECO terms satisfying the criteria laid out above.

When you read a sentence in the manuscript that you believe harbors a reference to an experimental method, you should use any of the following resources to identify the corresponding ECO term:

- ECO ontology browser [preferred -- note the brat server also lists the ECO terms we are currently using]
- EBI ontology browser
- BioPortal ontology browser

Note that you should always *try* to annotate to the *most specific* (child) term possible, given the information that is available *in the sentence*.

Once you have identified the term, select it in the region of text to annotate and use the pull-down list that will appear in BRAT to enter the annotation. See more the details on the BRAT manual.

Note: it might be more convenient for you to use a spreadsheet to note down the annotations that you identify in the text, or use the commenting function of Adobe Acrobat, and then use move to BRAT for perform the submission. But this is up to you.

### Commenting annotations

Annotation is a subjective process, where annotators try to assess what authors are stating in documents. To mitigate the subjective bias of annotators, each manuscript will be independently annotated by more than one annotator. This means that, eventually, annotations will have to be reconciled. We are still working out how we will perform this reconciliation. This process is greatly facilitated if you provide notes for your annotation that will enable you to answer any questions the person reviewing the multiple annotations may have in order to understand what motivated you to perform one annotation. In general, the less confident you are about an annotation, the more extensive your notes should be to enable the person performing the reconciliation to follow your train of thought.

### Troubleshooting the annotation

It may happen that the evidence is so loosely defined that you cannot really conclude where it should map to, or, on the other hand, that the evidence is properly defined, but you cannot find any corresponding term in ECO.

- For cases of ill-defined evidence:
  - Navigate the ontology upwards to identify a more generic evidence term that may be used to annotate the term.
  - If in doubt to the point at which it does not make sense even to make a *low confidence* annotation, please do not annotate the reference. The aim of the annotation process is on *quality, not quantity*.
- For cases of well-defined evidence without an obvious mapping in ECO:
  - ECO is a work in progress, and not all evidence is necessarily captured there. In fact, a substantial fraction of the ECO terms you will be using for annotation were created by undergraduates in the ErillLab working on CollecTF curation. If you firmly believe that the manuscript you are annotating contains a form of evidence not captured in ECO, please access the new term submission system in ECO and follow the instructions to submit a new term. In addition, you can mark the words in the article with the ECO:0000000 to signal that you mean for this to be evidence but there is no ontology identifier available yet. (This is explained in the BRAT manual)

## Annotation examples and trial run

Before embarking on the annotation process  it is important that you understand the nuances of annotating ECO terms in manuscripts. To do so, please first review the provided annotation examples. Before you are assigned your first manuscript, you will annotate a section of a manuscript and discuss the results with your peers.

## Double checking, comments and meetings

Manual annotation of scientific literature is not a trivial matter. Annotation is to a significant degree a subjective process, in which the curator assesses and interprets the scientific work reported in a manuscript. To further confound things, ontologies are not as neat and organized as they intend to be. Developed by the community, ontologies sometimes contain contradictory or ill-defined terms, and may be organized somewhat counter-intuitively. And, of course, figuring out whether references to ECO terms conform to the annotation guidelines (fully contained within sentence or sentence pair, with clear assertion made) can also be tricky.

## Double checking and comments

To avoid errors in curation, the standard in the field is for annotation to be performed independently by two or more curators (likely 3). Their annotations are then reviewed by a third party, or discussed through peer review. Here we will implement the peer-review system. Once the second curator for a manuscript completes the annotation, they will contact the team leader who will provide both curators with the annotations made by each curator. The team leader will then take notes of the discussion in order to inform the reconciliation of the annotation, detailing how the issue was resolved (e.g. "ECO:XXXXX was found to be more appropriate than ECO:YYYYYY because the context (Fig. XXX) shows that XXXX is used as evidence" or "Curators agreed that no annotation was required for this sentence").

### Comments on usage and terms, and meetings

While you are annotating, you will often find places where annotations may be difficult to call. You can sketch these issues in the comments section of BRAT, and take notes for yourselves, and bring them up during the regular curator meetings. You can also bring up problems that you have found in ECO (e.g. two terms apparently describing the same concept but with slightly different definitions or placements within the ontology), so that they are recorded and can be openly discussed at meetings in order to define a consensus over which terms to use in the future.
