# Supplementary material for: ECO-CollecTF: A Corpus of Annotated Evidence-Based Assertions in Biomedical Manuscripts
Source: Front Res Metr Anal. 2021 Jul 13;6:674205. doi: 10.3389/frma.2021.674205 (PMC8313968; doi:10.3389/frma.2021.674205)
Supplement: Supplementary file 7 [file DataSheet5.PDF]

KwCI simulation process

Parameter estimation

To perform simulations several parameters from the corpus had to be determined. We determined the density of annotated sentences in the dataset as the ratio between the total number of sentences with an annotation across all curators (for consecutive annotated sentences both sentences were counted as annotated) and the total number of sentences in the dataset.

We calculated the False Negative Rate (FNR) and False Positive Rate (FPR) for annotated sentences in our data. Each curation cohort was considered separately, with each curator in the group being evaluated against all other curators in the cohort. Unanimous agreement between all other curators was defined as the gold standard for assessing whether a sentence was a true positive (annotated) or negative (not annotated). Sentences that had disagreement were skipped. Using this reference, counts of True Positives (TP), True Negatives (TN), False Positive (FP) and False Negative (FN) were tallied for all curators to determine the corpus FNR and FPR.

Simulation

We developed a simulation procedure to examine the effect of ECO term selection disagreements between curators on the KwIC metric. In each simulation, we first generated a reference set of 1500 simulated sentences. For each sentence, the estimated annotation density (14%) was used to determine if the sentence was annotated. For annotated sentences, ECO terms were randomly drawn from the corpus distribution of ECO terms.

In each simulation, we then modelled the work of two curators performing the annotation process on the 1500 simulated sentences. Annotations were assigned to sentences following the annotated/non-annotated attribute of the reference and the corpus estimated FNR and FPR. To simulate disagreement on annotated sentences, one of the curators was assigned the reference ECO term and the other a term situated n hops away through ontological space, with the number of hops n defined by a random draw from a geometric distribution with success probability p.

This simulation process was repeated 100 times to generate 100 KwIC scores for a given probability of success for the geometric distribution. Simulations were run for the following values of p: 0.025, 0.050, 0.10, 0.25, 0.50, 0.75, and 1.0. The value of p for the corpus was estimated by tallying the term distance (hops) between curator pairs for annotated sentences.
